# Atomic layer deposition for fabrication of HfO_2_/Al_2_O_3_ thin films with high laser-induced damage thresholds

**DOI:** 10.1186/s11671-015-0731-8

**Published:** 2015-02-06

**Authors:** Yaowei Wei, Feng Pan, Qinghua Zhang, Ping Ma

**Affiliations:** Chengdu Fine Optical Engineering Research Center, Chengdu, Sichuan 610041 P. R. China

**Keywords:** Atomic layer deposition, Laser-induced damage threshold, Optical properties, 78.20.-e, 42.70.Hj, 42.25.Bs

## Abstract

Previous research on the laser damage resistance of thin films deposited by atomic layer deposition (ALD) is rare. In this work, the ALD process for thin film generation was investigated using different process parameters such as various precursor types and pulse duration. The laser-induced damage threshold (LIDT) was measured as a key property for thin films used as laser system components. Reasons for film damaged were also investigated. The LIDTs for thin films deposited by improved process parameters reached a higher level than previously measured. Specifically, the LIDT of the Al_2_O_3_ thin film reached 40 J/cm^2^. The LIDT of the HfO_2_/Al_2_O_3_ anti-reflector film reached 18 J/cm^2^, the highest value reported for ALD single and anti-reflect films. In addition, it was shown that the LIDT could be improved by further altering the process parameters. All results show that ALD is an effective film deposition technique for fabrication of thin film components for high-power laser systems.

## Background

The severity of environmental impacts due to energy generation and consumption increases the demand for improved clean and efficient energy production. Inertial confinement fusion (ICF) is a promising candidate for meeting future energy needs. Within the ICF system, massive optical components, such as mirrors and polarizers, are used to control the properties and directions of laser system beams responsible for the implosion process. These components must be fabricated both with high uniformity and a high laser-induced damage threshold (LIDT) over an aperture diameter greater than several tens of centimeters. In particular, the LIDT is a major constraint in the design of ICF optical components due to the high-fluence output and high-peak intensity of ICF laser systems [1].

Generally, due to the need for good environmental stability, optical coatings for such large and high-power laser systems are produced by electron beam evaporation [2]. However, this method is limited by the difficulty of depositing uniform and precise coatings over a large aperture. In order to obtain higher LIDT, technology such as laser conditioning was introduced. However, this led to further complications. Due to the challenges associated with the current high-power laser systems, generation of thin films with high laser damage resistance as well as high accuracy uniformity is an area of urgent focus.

Atomic layer deposition (ALD) is a promising method for obtaining thin films with both high laser damage resistance and high accuracy uniformity. ALD allows the microstructure of thin films to be determined by the deposition temperature, possibly allowing both high laser damage resistance as well as overcoming the self-limiting nature and lack of uniformity inherent in the chemisorption processes. In addition, ALD allows precise control of film thickness. Due to the high quality of films generated, ALD has a strong appeal not only for ICF systems but also for many applications such as semiconductor design, photoelectron research, solar power generation, and general optics [3-8]. To date, in-depth research on the laser damage resistance of thin films deposited by ALD is rare [9]. In this work, the key factors affecting the LIDT of thin films generated by ALD were investigated.

## Methods

Two groups of thin films for use in laser applications were deposited by ALD (R200, Picosun, Kirkkonummi, Finland). Each group contained single layers as well as an anti-reflector portion (contained two layers). Fabrication process parameters are listed in Table 1.

**Table 1:** **The fabrication process parameters in this work**

**Thin films (groups)**	**Deposition temperature (°C)**	**Precursor**	**Duration time (precursor A + purge + precursor B + purge)**
HfO_2_	300	HfCl_4_ + H_2_O	1.6 s + 5 s + 0.1 s + 1 s
		TEMAH + H_2_O	
Al_2_O_3_		Al(CH_3_)_3_ + H_2_O	0.1 s + 6 s + 0.1 s + 2 s
		AlCl_3_ + H_2_O	
HfO_2_/Al_2_O_3_		TEMAH + Al(CH_3_)_3_ + H_2_O/HfCl_4_ + Al(CH_3_)_3_ + H_2_O	Same as single-layer parameters
HfO_2_	250	AlCl_3_ + H_2_O	0.1 s + 6 s + 0.1 s + 2 s
			0.2 s + 10 s + 0.2 s + 6 s
Al_2_O_3_		HfCl_4_ + H_2_O	1.6 s + 5 s + 0.1 s + 1 s
			1.6 s + 10 s + 0.1 s + 2 s
HfO_2_/Al_2_O_3_		TEMAH + AlCl_3_ + H_2_O	Same as single-layer parameters

**Figure 1:**
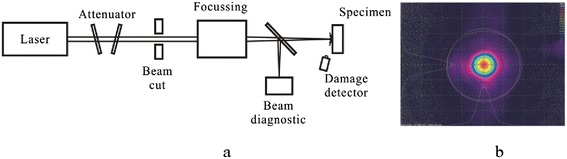
**Experimental setup and laser beam profile. (a)** Laser damage testing bench; **(b)** Typical beam profile at sample position.

Optical properties such as the absorption, refractive index, and transmission spectra were analyzed in detail. For thin films designed for laser applications, the most important property is the LIDT. Therefore, the LIDTs were measured for comparison using a Nd:YAG laser in 1.000 on 1 mode (ISO 21254) [10], at a wavelength of 1,064 nm, a pulse duration of 3 ns (FWHM), and a pulse frequency of 100 Hz. The experimental setup and laser beam profile are shown in Figure 1. The laser was focused to provide a far-field circular Gaussian beam with a diameter of 300 μm at 1/e^2^ of the maximum intensity. The angle of incidence was close to 0°. A Si-photodiode was used to monitor the scattered light variation and served as an online detection system. The damaged sites were reconfirmed offline using a Nomarski microscope with magnification ≤ ×200.

Different pulse numbers were used to complete various S-on-1 damage tests (ISO 21254) [10]. Once damage had occurred, subsequent pulses were cut off by the on-line damage-detection system and moved the sample to the next site. To study the endurance and lifetime of the samples, the damage threshold was calculated according to Equation 1, where *H*_*th(N)*_ represented the damage threshold of S-on-1 test using *N* pulses, and Δ was given by the intersection of the tangent at the point (1, *H*_*th,1*_) and the constant level *H*_*th,∞*_.

1 $$ {H}_{th\;(N)}={H}_{th,\infty }+\frac{H_{th,1}-{H}_{th,\infty }}{1+\frac{1}{\Delta} ln(N)} $$

Finally, damage morphologies and film cross-sections were analyzed using dual beam microscope (focus ion beam and scanning electron microscope, FIB/SEM) and atomic force microscope (AFM).

## Results and discussion

**Figure 2:**
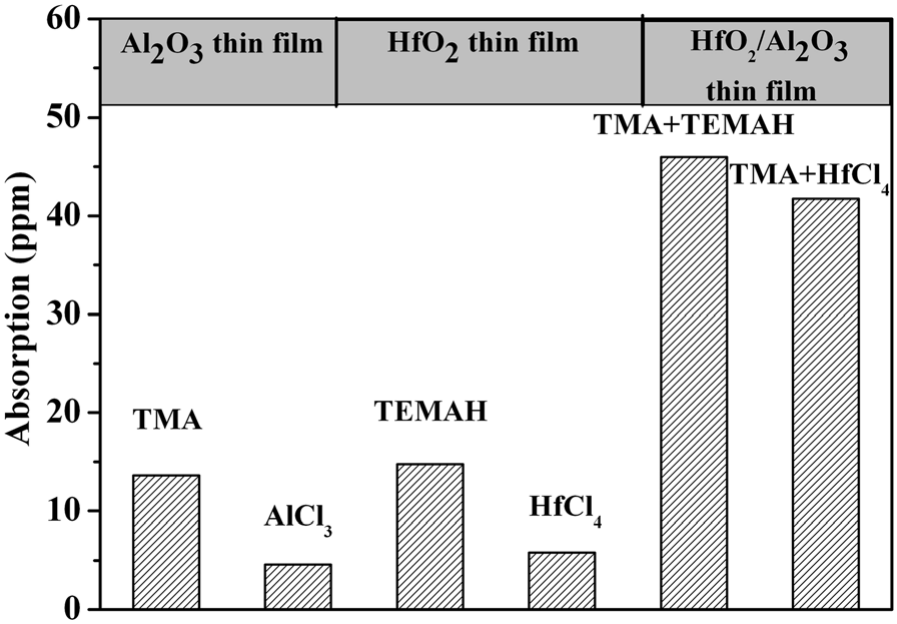
**Results of absorption measurements for thin films deposited with various precursors.** TMA, trimethyl aluminum; TEMAH, tetrakis (ethylmethylamino) hafnium.

Figure 2 shows absorption values for the thin films deposited using a variety of precursors. Specific precursors used for each thin film were listed on top of each column in the Figure 2. Trimethyl aluminum and tetrakis (ethylmethylamino) hafnium were abbreviated as TMA and TEMAH, respectively. In addition, due to the low vapor pressure of precursors such as AlCl_3_ and HfCl_4,_ hot sources were needed to aid the deposition process. Because the coating instrument had only a single hot source, the anti-reflector was deposited using pure organic precursors and in combination for the inorganic/organic precursors. The thickness of the Al_2_O_3_ and HfO_2_ single layers were both 100 nm. For the anti-reflector, the thickness of the Al_2_O_3_ and HfO_2_ films were 199 nm and 79 nm, respectively. As shown in Figure 2, single layers deposited with the inorganic precursor had smaller absorption values than those deposited using an organic precursor. The absorption value for the anti-reflector film was greater than the single layer due to the increased film thickness. In addition, the anti-reflector deposited using pure organic precursor had a larger absorption value than anti-reflector deposited in combination with the inorganic/organic precursors. These absorption results show that precursor type has a large affect on the absorption of the thin film. When the organic precursor was used, the adsorption force was larger than that for an inorganic precursor. This was evidenced by the fact that although the deposition process was carried out for the same duration of time, a greater number of organic groups remained in the film as compared to inorganic groups. Organic groups showed intense absorption for infrared laser wavelength. Overall, single-layer thin films deposited using the organic precursor had larger absorption values than those fabricated using an inorganic precursor.

**Figure 3:**
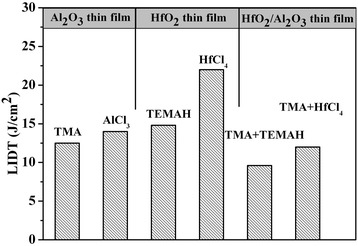
**LIDT result for thin films deposited using different precursors.**

Figure 3 shows the LIDT results for films deposited using different precursors. For Al_2_O_3_ and HfO_2_ single-layer films, the LIDTs for films deposited using inorganic precursor (14 J/cm^2^ and 22 J/cm^2^, respectively) were larger than those for films deposited using organic precursor (12.5 J/cm^2^ and 14.7 J/cm^2^, respectively). For the HfO_2_/Al_2_O_3_ anti-reflector films, those deposited with pure organic precursor had lower LIDT values (approximately 8 J/cm^2^). Taking into consideration the absorption results, the LIDT results might be explained as follows. When the organic precursor was used, adsorption force was larger as compared to when the inorganic precursor was used. Because the same deposition time used for all films, a greater amount of organic groups remained in the film as compared to inorganic groups. Previous results showed that the organic groups had an intense absorption in the IR. When the laser source was used in a high-energy mode, the dominant damage morphology was due to absorption induced peeling. Therefore, the precursor types did have an effect on the LIDT of thin films subjected to a high-energy laser. Therefore, thin films deposited using inorganic precursor can result in a higher LIDT as compared to films made using organic precursor.

**Figure 4:**
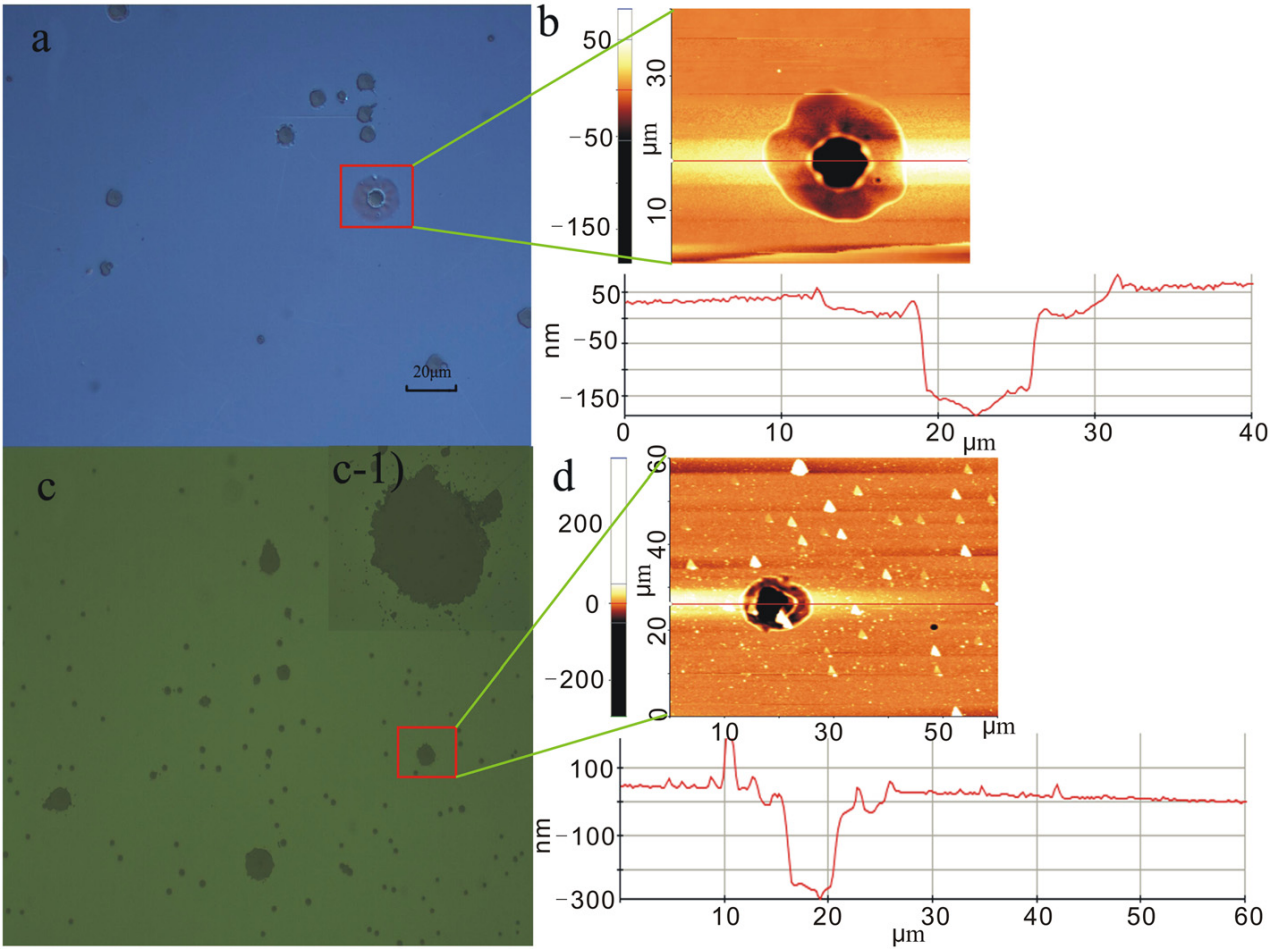
**Damage morphologies for HfO**_**2**_ **/Al**_**2**_ **O**_**3**_ **anti-reflector deposited using the different precursor types in group two. (a**, **c)** Damage morphologies for anti-reflector deposited with HfCl_4_ and TMA or TEMAH and TMA, respectively, obtained using a Nomarski microscope. **(b**, **d)** Damage spot images obtained using AFM.

Analysis of the damage morphology was of particular interest. In this work, damage morphologies were observed using a Nomarski microscope in combination with AFM. Figure 4 shows the damage morphologies for the HfO_2_/Al_2_O_3_ anti-reflector film deposited with either inorganic or organic precursors. Figure 4a shows the damage morphology for the anti-reflector deposited using precursor HfCl_4_ and TMA, and Figure 4b shows the AFM image for the damage spot seen in Figure 4a. A crater-like structure in combination with peeling is the dominant damage morphology. Using the image shown in Figure 4b, the depth of the damage spot was measured to be approximately 200 nm, equal to the thickness of Al_2_O_3_ portion of the thin film. This is evidence that when the HfO_2_/Al_2_O_3_ anti-reflector was deposited with precursor HfCl_4_ and TMA, subsequent laser-induced damage only occurred only in the Al_2_O_3_ layer. The Al_2_O_3_ thin film was evidently the vulnerable component of the HfO_2_/Al_2_O_3_ anti-reflector. Figure 4c shows the damage morphology for the anti-reflector deposited using pure organic precursor, and Figure 4d shows the AFM image for the damage spot in Figure 4c. Figure 4(c-1) displays a magnified image for the damage spot indicated by a red circle. When the pure organic precursor was used, peeling (as seen in Figure 4c) became the dominant damage morphology for the HfO_2_/Al_2_O_3_ anti-reflector. The damage depth was calculated using the image shown in Figure 4d and was seen to be equal to the thickness of the entire anti-reflector (approximately 270 nm). This result indicates that the complete anti-reflector sustained damage as opposed to damage occurring only to a single layer. These results lead to the clear conclusion that precursor type can affect the film LIDT.

**Figure 5:**
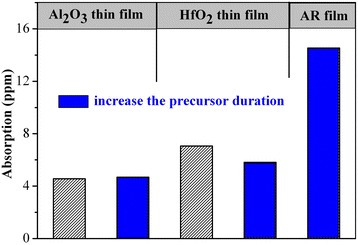
**Absorption values for thin films before and after increasing the precursor duration time.**

Figure 5 shows the film absorption values before and after increasing the precursor duration time (precursor deposition cycle time). For the Al_2_O_3_ thin film, the absorption showed little change, with the absorption level remaining low. In contrast, the HfO_2_ absorption became smaller after increasing the precursor deposition cycle time.

**Figure 6:**
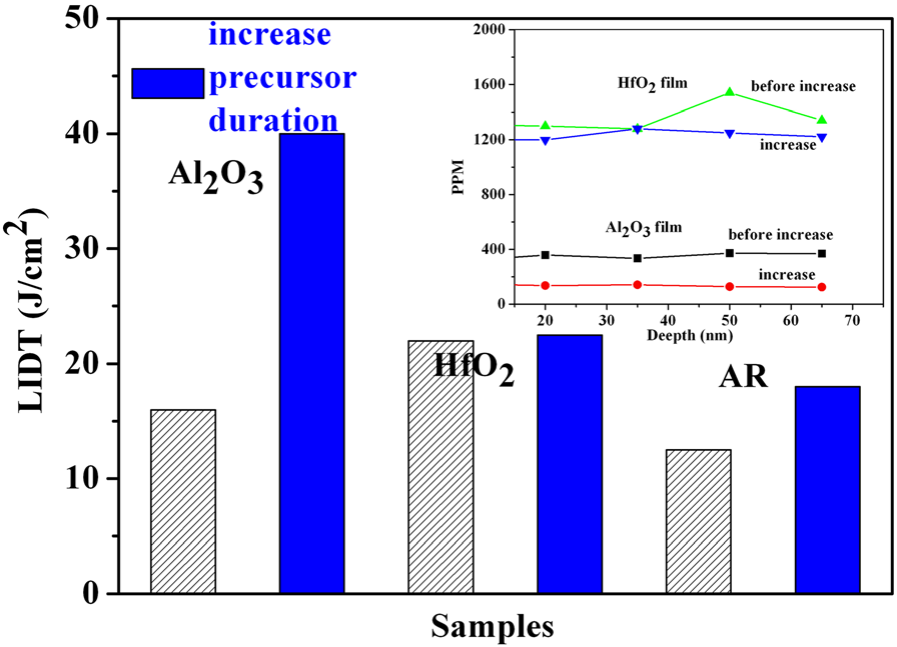
**LIDT results for thin films before and after increase the precursor deposition cycle time.**

The LIDT results for the thin films before and after increased precursor deposition cycle time are shown in Figure 6. The inset in Figure 6 shows the amount of residual organic elements in the single layer before and after the increased precursor deposition cycle time. As can be seen, the LIDT was greatly improved for the Al_2_O_3_ thin films (from 15 J/cm^2^ to 40 J/cm^2^) but showed little change for both the HfO_2_ thin films (from 21 J/cm^2^ to 22 J/cm^2^) and HfO_2_/Al_2_O_3_ anti-reflector (from 11 J/cm^2^ to 18 J/cm^2^). The changes to deposition conditions included decreasing the deposition temperature (from 300°C to 250°C) and increasing the precursor deposition cycle time. The temperature change from 300°C to 250°C did not significantly alter the microstructure for the thin films. However, when the precursor deposition cycle time was increased, the chlorine element was decreased for both the Al_2_O_3_ and HfO_2_ single layers. It is therefore most likely that the LIDT change can be attributed to the precursor deposition cycle time. An increase in the pulse duration did not significantly disrupt the chemisorption process, and reactions were carried forward sufficiently. In addition, organic residual in the films due to incomplete reactions was noticeably decreased. Unfortunately, organic residual remained the weak point in the films used in the laser system. Upon increase of the pulse duration, the residual chlorine element within the Al_2_O_3_ thin films decreased from approximately 400 ppm to approximately 200 ppm, and the LIDT showed a large change. In the case of the HfO_2_ thin films, both the residual chlorine element and the LIDT showed little change. Specifically, upon increase of the pulse duration the residual chlorine element maintained a high level (approximately 1,400 ppm), meaning that thermal damage due to chlorine absorption within the film was the dominant force leading to film damage. This conclusion was also confirmed by the damage morphology results.

Furthermore, the LIDT of the anti-reflector was able to attain a high level comparable to that of the HfO_2_ thin film. This may be due to the HfO_2_ thin film being ‘short plank’ within the anti-reflector. Therefore, improving the LIDT of the HfO_2_ film could improve the LIDT of the anti-reflector.

**Figure 7:**
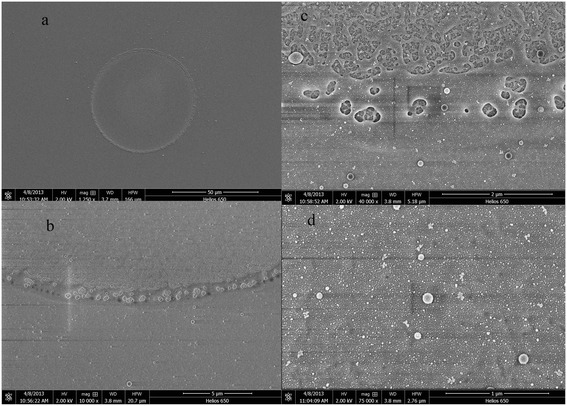
**SEM images of Al**_**2**_ **O**_**3**_ **thin film damage morphology. (a)** Damage spot. **(b**, **c)** Magnified image of the bottom for the damage spot with different magnification levels. **(d)** Surface morphology of the Al_2_O_3_ thin film.

Figure 7 shows SEM images of the damage morphology of an Al_2_O_3_ thin film. Figure 7a shows the damage morphology in the free field. The laser damage spot exhibited a ring shape. Figure 7b shows a magnified image of the damage spot ring. The image shown in Figure 7c gives evidence that the damage spot was made from fused ejection. Figure 7d shows the Al_2_O_3_ film configuration. As shown in Figure 7a, the damage spot on the Al_2_O_3_ thin film exhibited a standard circular shape, which is evidence that the film has a higher damage threshold. Investigation of damage morphologies showed that the damage spot had experienced melting but not peeling; evidence that the film adhesion was robust and the dominant damage mechanism might not be due to thermal effects.

**Figure 8:**
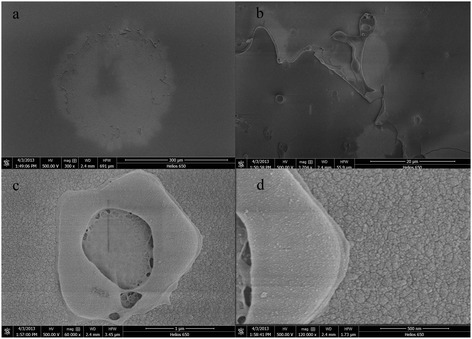
**SEM images for HfO**_**2**_ **thin film damage morphology. (a)** Damage spot. **(b)** Magnified image of the edge of the damage spot. **(c**, **d)** Magnified images around the damage spot with different magnification.

Figure 8 shows the damage morphology of the HfO_2_ thin film. The dominant damage morphology was seen to be peeling when the laser source was used at a low energy (as shown in Figure 8a,b). This observation leads to the conclusion that a larger mechanical force was produced during the damage process, in contrast to the damage morphology seen for Al_2_O_3_ thin films. From this analysis, it is clear that the dominant damage mechanisms for HfO_2_ and Al_2_O_3_ thin films are different. Further damage morphology can be seen in Figure 8c,d, where small damage spots could be seen in the black area around the main damage spot. When the damage spot was magnified only slightly, the small damage spots were not observed. When a higher level of magnification was used, the damage spots looked as if they were fused. These damage spots may show the original stages for the damage process: the film only appears to melt, not a small process in itself. In addition, the film surface morphology obtained from Figure 8d exhibited an obvious cluster. Compared to the smaller clusters for Al_2_O_3_ thin film, the larger clusters on the HfO_2_ film surface might be due to the film crystallization.

**Figure 9:**
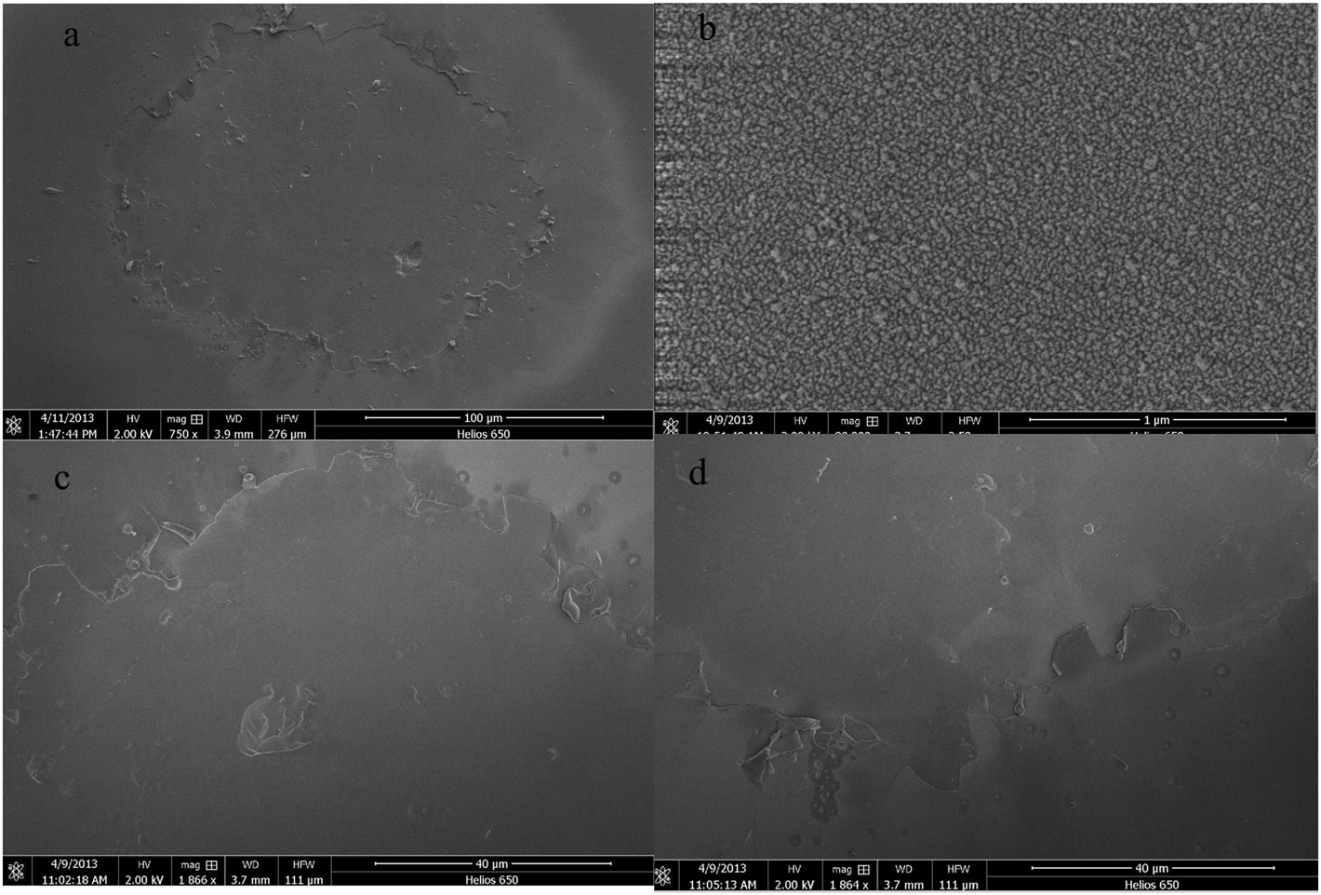
**SEM images for HfO**_**2**_ **/Al**_**2**_ **O**_**3**_ **anti-reflector damage morphology. (a)** Damage spot. **(b)** Surface morphology of the anti-reflector. **(c**, **d)** Magnified image of the edge of the damage spot.

The HfO_2_/Al_2_O_3_ anti-reflector damage morphology was shown in Figure 9a and the feature was also amplified in Figure 9c,d. In addition, Figure 9b shows the film surface morphology. As shown in Figure 9, the damage morphology of the HfO_2_/Al_2_O_3_ anti-reflector was similar to that of the single HfO_2_ layer. The dominant damage morphology was observed to be peeling. The former results showed that the LIDT of the HfO_2_ single layer and the HfO_2_/Al_2_O_3_ anti-reflector were small and the LIDT of the Al_2_O_3_ film was much higher. It can therefore be concluded that the HfO_2_ layer was the damage-inducing factor for the HfO_2_/Al_2_O_3_ anti-reflector. Therefore, improving LIDT of the HfO_2_ films could further improve the LIDT of the anti-reflector.

## Conclusions

Single-layer and anti-reflector thin films were prepared using ALD with various deposition process parameters such as differing precursor types and pulse durations. Optical and damage properties were subsequently analyzed. Precursor types were shown to affect the film absorption and the final level of induced damage that occurred. When the pulse duration was increased, films LIDTs were greatly increased for Al_2_O_3_ single-layer films, while only slightly increased for HfO_2_ single-layer and HfO_2_/Al_2_O_3_ anti-reflector films. Specifically, The LIDT of the Al_2_O_3_ thin film reached 40 J/cm^2^. The LIDT of the HfO_2_/Al_2_O_3_ anti-reflector film reached 18 J/cm^2^, the highest known value for ALD single and anti-reflect films previously reported. In addition, it was shown that the LIDT could be improved via altering the processing parameters. All results showed that ALD is an effective film deposition method for the fabrication of components for use in a high-power laser system.

## Abbreviations

ALD: atomic layer deposition
ICF: inertial confinement fusion
LIDT: laser-induced damage threshold
TEMAH: tetrakis (ethylmethylamino) hafnium
TMA: trimethyl aluminum
